# Unnecessary Pain in Dental Operations

**Published:** 1893-03

**Authors:** James A. Reilly


### UNNECESSARY PAIN IN DENTAL OPERATIONS.[1]

BY JAMES A. REILLY, D.M.D.

It is not my intention this evening to attack any established theories or to attempt to overthrow any cherished opinions or prejudices. I simply desire to call your attention to a few points in every-day practice, and would prefer to suggest a few things you ought not to do rather than those you should do. Indeed, I am to address you from the stand-point of a patient in the chair rather than as the operator at its side.

One forenoon during my senior year at the Harvard Dental School, and while in charge of the dental department of the Bennet Street Dispensary, among the numerous patients was a lad of twelve or thirteen years. He went through the usual preliminaries required in order to have an inferior bicuspid tooth extracted. The operator mechanically picked up his mirror and pliers to examine the tooth, or what remained of it, and almost simultaneously with their introduction into the boy's mouth there was a terrific scream and a plunge that almost carried him through the window. An attempt at extraction by a street dentist had resulted in the removal of the crown, leaving the entire coronal portion of the pulp standing unprotected. The dentist simply plunged his pliers into the mass of living tissue. Was not that an abuse utterly reprehensible on his part? I think it was, and so would you, I believe, had you been the sufferer. Yet we are doing just such things every day in one form or another.

That "familiarity breeds contempt" is nowhere more noticeable than in the use of dental instruments and appliances. Not long since, a gentleman somewhat prominent in dental organizations told me he had not a dozen excavators in his possession; that he excavated all his cavities with the aid of the dental engine, and wished to wager me that I could not find a cavity in a tooth that

---

[1] Read at a meeting of the American Academy of Dental Science held in Boston, December 7, 1892.

he could not reach and prepare as well, if not better, with the engine than it could be accomplished with hand-excavators. Upon being questioned if his patients did not complain of being hurt, he replied, "Confound the patients! my duty is to protect myself." If this gentleman could but be patient and operator at the same time, I have no doubt that he would be easily induced to trade some of his burrs for hand-excavators. Has he not, to say the least, become too "familiar" with his engine? This I consider an extreme case of abuse, for, allowing for a moment that all cavities may be reached (which I do not believe, unless he destroys a vast quantity of sound tooth-substance), the time that is gained by its use is but a trifling compensation for the torture that is thus inflicted on children and excessively nervous adults, and I suppose he has such patients. He may run his engine slowly, use the sharpest burrs and all the obtundents at his command, but does he diminish the loss of tooth-structure thereby, or reduce the inherent antipathy to dental operations which the average patient has? Does he not absolutely destroy the last vestige of confidence the little one may possess who has been beguiled into the chair by its parent with the unqualified assurance that "it will not hurt a bit"?

Another appurtenance, no less barbarous in some of the details than the untimely use of the dental engine, is the rubber dam. A prominent writer says, if it is at all difficult to apply, the rubber dam should not be used in the cases of the very young, very sensitive, or very nervous patients. How many of us draw the line at these classes? It is not my intention to point out the occasions for its use or to urge upon you its abandonment, for I consider it a *sine qua non* to good results in numberless cases. But I would like to call your attention to the contempt for your patients' feelings that a "familiarity" with its application breeds.

You are all aware how quickly you jerk your head away if by accident the floss slips too rapidly between your own teeth and burrows itself in your gums while you are cleansing them. How often the same thing is perpetuated on your patients, and nothing thought of it, by you at least, while you are laying coil after coil of cable on teeth that oftentimes do not require ligatures! Frequently, indeed, they serve only to obstruct access to the cavity. We all know, or should know, that with holes of proper size and shape in the dam the employment of ligatures is necessary only in a limited number of cases, provided the tartar has been removed from about the margins of the gum. But for pure, unalloyed torture, permit me to present to your consideration a clamp and an awkward or heavy-handed operator, and I think there are a few such in the profession.

I speak from experience, for it once fell to my lot to sit in the chair with a clamp on an inferior wisdom-tooth, compelling me to keep open house during the space of three and one-half hours. My knowledge now teaches me that it was entirely unnecessary, and that the cavity might have been filled, with the aid of napkins, in less time than it took to get the rubber and clamp adjusted, and with infinitely less pain and discomfort.

Now, I do not maintain that the clamp should be relegated into "innocuous desuetude," but I do say that extreme care should be exercised in selecting the proper ones to be used in each particular case, so that they may be easily adjusted, and that the most delicate and extreme accuracy of manipulation possible be employed while placing them upon the teeth. I know of nothing more repellant to the average patient than the rubber dam and its accompaniments; therefore I think it behooves us to manifest a little compassion by dispensing with the use of the clamp, or the ligature, and even the dam itself, whenever it is practicable.

Another medium for pain-culture, and one which gives ample opportunity for the application of all the reserve abuse we may have stored away, is obtained during a course of regulating. A great deal of pain and soreness of course, it is needless for me to say, is unavoidable while moving the teeth about, but there is also a large amount carelessly inflicted by over-anxious operators, too eager to accomplish in one day what should take a week, and again, doing to-day what they must undo to-morrow.

I once saw a case of regulating that was worthy of the attention of the society for the suppression of cruelty to children. The teeth were very much displaced, and appliances were adjusted to almost all the teeth simultaneously. Too much force was applied, and too long an interval allowed to elapse before changing, so that when I saw the mouth there was scarcely a tooth in the superior maxilla that could not have been easily removed with the fingers. For articulation the patient could not bring the teeth together without suffering intense pain. And all this under the direction of a reputed skilful operator. The effect of such operations is most pernicious, for the impression they produce on the patient's mind is often more enduring than what they effect in the physiognomy, and frequently nothing short of an exposed pulp will permit further dental operations during those years when the closest scrutiny and care should be exercised.

This, then, is the point I wish to make regarding the lack of care to avoid pain during regulating: that oftentimes nothing is gained by the operation, because if you succeed in holding your young patient's interest to a successful termination of the work you have also generated mentally such an intense dread and abhorrence of you and your benefactions, that it is not until caries has obtained a firm foothold, and sometimes even demolished that which for months engaged all your energies to perfect and beautify, that your ministrations are again solicited. Would it not be more preferable to "make haste more slowly," and retain the confidence of the little ones, even at the cost of not accomplishing quite as much as you would wish to do at that time? This same principle is equally applicable to the filling of young teeth, and I frequently do nothing more at the first sitting than to cleanse a few teeth with the stick, or wipe out a cavity with an antiseptic and insert a little gutta-percha or cement, sometimes without removing any decay whatever. For I consider my time well employed if I can succeed in dispelling this dread which always possesses them at the first sitting.

There are many minor things in our routine work that might be dilated upon in a paper of this kind which are really painful, although to us they seem very trifling, and if our patients shrink from them we are prone to ascribe it to fear or timidity, when we really are inflicting pain. By the habits of some dentists one would suppose the patient had no rights that the dentist should respect. He lolls over and leans on his patient, making of their head a cushion and support for his arm till the patient is well-nigh exhausted. It does not diminish the discomfort any to know that it is sometimes done unconsciously. That much inconvenience and unnecessary pain are caused by our neglect to scrutinize our processes and individual peculiarities, or by failure to keep them before our eyes, is not to be denied. Is not unnecessary pain frequently caused while putting on gold caps, bridges, and collars for crowns, without first applying cocaine to the gum margin? Is it not unnecessary pain to continue nibbling at an exposed pulp that had not wholly succumbed to the arsenious paste? I think you will agree with me that to catch the lip beneath the thumb while making it a fulcrum against the teeth is rather painful, and that to wash out a cavity with cold instead of tepid water may produce avoidable pain.

How common an experience it is to hear an outcry, or see a twitching of the head and body immediately upon using the chip-blower while excavating! It does not take place so much if we use warm air. Yet, do we always use it? Is it not positively abusive to whack away at a tooth for hours with the automatic mallet, when hand-pluggers might be used with so much more comfort, at least during the first part of the filling? Is it not an abuse to inflict quick wedging as ordinarily performed? Is it not an abuse, in taking full impressions for artificial dentures, to overflow the plaster from your impression-cup into the throat of your patient, when a smaller quantity would produce a much better result by giving a more accurate impression, because the parts are not so likely to be disturbed by retching and coughing? Is it not abusive for a dentist having a strong, muscular hand, with a heavy touch and a vise-like grip, to rush and hurry through his work as if he were under the impulse of electricity? My observations lead me to believe that rapid operators hurt more than slow ones. I believe that after a fair rate of speed has been attained, any acceleration of it is obtained only at the expense of delicacy of touch and of the patient's nervous system.

The conclusions I drew from my experience as a patient was, that more pain and discomfort arose from outside influences, if I may so term them, than from the actual preparation of the tooth to be filled. It is within the ability of everybody to cultivate a delicacy of manipulation, if they do not naturally possess it, and delicate manipulation is a powerful factor in dispelling the dread so universal in the minds of the people relative to dentistry. As President Elliot said the other day at the meeting in behalf of Harvard's new dental school, "It is the dread of pain which makes people miserable."

